# Supplementary material for: An Assessment of Health Behaviours in Primary Care Patients: A Cross-Sectional Study
Source: Healthcare (Basel). 2024 Jul 15;12(14):1405. doi: 10.3390/healthcare12141405 (PMC11275404; doi:10.3390/healthcare12141405)
Supplement: Supplementary file 1 [file healthcare-12-01405-s001.zip › healthcare-3036382-supplementary.pdf]

An assessment of health behaviours in primary care patients. A cross-sectional study

Supplementary Material

Table S1. Predictors for total HBS score – multivariate linear regression

p for the model= 0.000001 R^2= 0.178821	**B**	**SE for B**	**-95% CI**	**+95% CI**	**p value**
Sex [Male] - Reference category	-	-	-	-	-
Sex [Female]	6.82281	1.350524	4.160035	9.485585	0.000001
Age	1.207982	0.716479	-0.204671	2.620636	0.093325
BMI	-0.35662	0.151901	-0.656117	-0.057124	0.019846
Education [Primary + vocational] - Reference category	-	-	-	-	-
Education [Secondary]	0.552953	1.884357	-3.162359	4.268265	0.769481
Education [Higher]	2.282487	2.044999	-1.749559	6.314532	0.265679
Residence [Village] – Reference category	-	-	-	-	-
Residence [City up to 50 000 inhabitants]	-2.165807	2.397145	-6.892164	2.560551	0.367329
Residence [City above 50 000 inhabitants]	2.424217	4.214249	-5.884852	10.733286	0.565761
Residence [City above 200 000 inhabitants]	0.203063	1.670746	-3.091082	3.497207	0.903383
Economic status [Very bad] - Reference category	-	-	-	-	-
Economic status [Bad]	-3.207225	6.129246	-15.29202	8.877569	0.601357
Economic status [Average]	2.549823	5.550323	-8.393532	13.493179	0.646435
Economic status [Good]	4.900067	5.598541	-6.138356	15.93849	0.382473
Economic status [Very good]	2.08092	5.859758	-9.472535	13.634376	0.722866
Number of health conditions	-0.289583	0.689279	-1.648607	1.06944	0.674836
Hypertension [No] - Reference category	-	-	-	-	-
Hypertension [Yes]	-0.578502	1.652873	-3.837407	2.680402	0.726701
Diabetes [No] - Reference category	-	-	-	-	-

Diabetes [Yes]	-0.082623	2.044551	-4.113785	3.948539	0.967805

Table S2. Predictors for HBS domains – multivariate linear regression

	B	SE for B	-95% CI	+95% CI	p value
Individual preventive behaviours<br>p for the model=0.269761<br>R^2= 0.013863					
Hypertension [No] - Reference category	-	-	-	-	-
Hypertension [Yes]	-0.21502	0.104338	-0.420738	-0.009301	0.04059
Health behaviours related to diet<br>p for the model=0.000556<br>R^2=0.109883					
Sex [Male] - Reference category	-	-	-	-	-
Sex [Female]	0.232193	0.086061	0.06251	0.401876	0.00756
BMI	-0.02697	0.00968	-0.046055	-0.007885	0.005836
Health behaviours related to physical activity<br>p for the model=0.000001<br>R^2=0.182059					
BMI	-0.05706	0.012627	-0.081956	-0.032165	0.000011
Unhealthy behaviours<br>p for the model=0.000113<br>R^2=0.128793					
BMI	-0.030278	0.009309	-0.048632	-0.011924	0.001338
Education [Primary + vocational] - Reference category	-	-	-	-	-
Education [Secondary]	0.17933	0.115479	-0.048355	0.407015	0.12199
Education [Higher]	0.300733	0.125323	0.053638	0.547827	0.01731
